# Failure probability and location identification of damaged insulators using normal function and monitored leakage current

**DOI:** 10.1371/journal.pone.0314708

**Published:** 2025-06-11

**Authors:** Moein Monemi, Seyed Mohammad Shahrtash, Mohsen Kalantar

**Affiliations:** Center of Excellence for Power System Automation and Operation, School of Electrical Engineering, Iran University of Science and Technology, Tehran, Iran; SRM-RI: SRM Institute of Science and Technology (Deemed to be University) Research Kattankulathur, INDIA

## Abstract

The main task of insulators is to isolate the conductor from the tower. These insulators must be able to isolate the high voltages of the transmission lines from the tower without having a leakage current (LC). These insulators get damaged over time and may not work properly. Currently, identifying and replacing defective insulators, in addition to being time-consuming, requires frequent and long blackouts to be imposed on the customers. For this reason, this paper examines the problems and failures caused by the use of insulators in networks with different voltage levels and suggests the failure rate for planning the maintenance of these electrical network equipment. Based on this, according to the insulator leakage current data, the failure or damage rate of the insulator equipment will be investigated using the normal distribution. This distribution function will calculate the probability of insulator failure using LC data, and then the failure rate and the priority of insulator maintenance will be measured compared to other data. According to this article, the final goal of the proposed methodology will be to determine the failure rate as well as decision-making for the maintenance of the insulator equipment and finally determine the damaged insulator among all the insulators of the electrical network. By identifying the insulators with a higher failure rate, the maintenance team will decide to repair or replace the damaged insulator before the insulator failure and the fault in the power grid.

## 1. Introduction

One of the important parts of power transmission systems is the insulation of transmission lines, so that the efficiency of transmission systems is related to the health of insulation systems. Therefore, technical defects in the used insulators are important [1]. One of these defects is the presence of impurity in the insulator building, which can be known as partial discharge (PD) source. In addition to the phenomenon of puncture in insulators, factors such as mechanical failure, arcing, etc. are among the other factors that cause the failure of ceramic insulators in the distribution and sub-transmission network. Also, pollution deposition is the main source of leakage currents (LC) and this leads to discharge activities on the surface of insulators. The reason for this claim is the high number of failures recorded due to the existence of problems in insulators [1]. It is also necessary to mention that outdoor failures caused by the contaminant deposition, dust, vandalism and birds’ nests also include a major part of the failures in insulators, and therefore, it is very important to consider the environmental conditions in the monitored data of the insulator and to diagnose the failure [2,3].

Currently, the existence of defects and impurities in insulators is one of the important factors in the occurrence of phenomena such as insulator puncture. Accurate and timely identification and location of these defects, before such incidents occur in electricity distribution and sub-transmission networks, helps a lot to significantly reduce blackouts.

One of the effective methods to diagnose the health of insulators is extracting characteristics from its LC waveform [1]. The reliable insulation level provided by an insulator depends on the amount of LC that flows on the surface of the insulator. When the LC of an insulator increases, it indicates that a higher amount of current is flowing along the surface or through the insulation material rather than through the main conductive path. This current, while usually small, can lead to the buildup of voltage at the insulator’s terminal, resulting in overvoltage. So, increasing the LC of the insulator causes overvoltage on the insulator terminal. This voltage can be between 1000-5000 volts depending on weather conditions. For instance, during high humidity, fog, or rain, the surface of the insulator may become partially conductive, increasing the LC and thereby raising the voltage across the terminal.

This higher voltage contributes to network losses in several ways. First, leakage currents convert a portion of the electrical energy into heat, causing resistive losses, which reduce overall network efficiency. In addition, repeated exposure to overvoltage stresses the insulation material, degrading its integrity over time. This degradation can increase the risk of partial discharges, which are small, localized discharges within the insulation that can gradually weaken it, leading to insulation breakdown, potential faults, and increased maintenance requirements. Consequently, these losses and potential failures not only impact network reliability but also increase operational costs.

For this purpose, LC must be reduced in an acceptable range. Therefore, measures such as regular periodic visits, periodic washing of insulators are carried out to reduce the pollution of the insulator surface and other factors that affect the size of the LC.

Apart from the discussion of the threats of the increase in the LC of the insulator, which leads to an increase in the rate of accidents and a decrease in the level of reliability in the continuity of electricity supply, this current can be seen as a practical tool and a suitable opportunity for monitoring the working condition of an insulator in different conditions. Valuable reviews and information include checking the surface of the insulator in terms of surface pollution, the amount of electrical and mechanical stresses applied to it, the health status of the insulator and in general the performance and efficiency of the insulators in their designs and the materials used in its construction [4]. This tool is used in all kinds of tests, laboratory samples and real samples in field tests. In monitoring the LC, in order to test different types, insulators installed in the substation, anti-pollution and anti-fog insulators, insulators with an insulator or on an insulator chain, etc. have been implemented [5].

In [4], Positron Company unveiled a device called “Positron” whose work is based on measuring the electric field around the insulator. By using this device, valuable information about the health status of the insulator was obtained.

Deep learning is known as structured deep or hierarchical learning. They are part of a larger family of learning methods based on learning data representations. The researchers conducted in the last decade about monitoring the condition of insulators pay special attention to the use of image processing methods and deep learning topics [6]. Reference [7] considers the correct identification of the insulator from other details in the images prepared of the lines and towers as an important principle in identifying the presence of cracks and physical defects in the insulators. This index becomes important due to the fact that the insulators have different dimensions according to the application and the voltage level used, and during the preparation of images, the lines are recorded at different angles in the image, which makes it difficult to identify failures.

Region-based Convolutional Neural Networks (R-CNN) provides a high-accuracy object detection method using deep convolutional neural network (NN) for object classification, which is discussed in [8]. Due to the problems in the R-CNN, the use of faster NNs called Fast R-CNN and Faster R-CNN is suggested [9] and [10]. In accelerated R-CNN, instead of running the NN on 2000 parts of an image, the image is fed to the neural network only once. With this work, the NN creates convolutional feature maps and by using these maps, the suggested parts can be extracted.

Aerial photographing and image processing of insulator chain using R-CNN has been improved and faster, in order to identify three types of insulators and insulator cap failure, taking into account the effect of the distance of photographing the insulator chain, it has been seen in [9]. Identification of defective insulators using an aerial image prepared with a complex background and many details along with the introduction of a two-level deep learning hybrid method based on R-CNN has been introduced in [10]. In this type of aerial images, finding a small defect in the insulators used in the lines is a very challenging task. In [11], a new method for detecting insulation defects from both global and local levels is presented, which improves the speed of R-CNN for simultaneous detection of insulation and related defects in the entire image. On the other hand, all existing insulators are extracted and considered as input to perform further inspection in each image at the pixel level.

According to [12], by using image processing, it has identified the damages on the surface of the insulator. According to this reference, the detection rate of the burn mark algorithm is 80.43%.

In [13], the effect of uneven distribution of pollution in conditions of different humidity on insulator has been measured. Also, according to this reference, an Artificial Neural Network (ANN) has been used to predict the flashover voltage.

Authors in [14] and [15] have introduced a procedure to determine the failure of the monitored data of the circuit breakers, which is general and can be used for other electrical network equipment. A similar procedure has been used in [16] to diagnose the failure and functional age of gas pipeline equipment.

In wet and windy conditions, conductive contaminants begin to dissolve in water on the surface of the insulator, which increases the leakage current and affects the performance of the insulator. According to [17], it uses a data collection system to measure the insulator leakage current and weather parameters around the insulator. Artificial intelligence is then used to create a predictive model for leakage flow based on weather parameters. Then this information is transferred to maintenance users. Also, in [18], the formulation of the resistance of the residual pollution layer on the insulator is proposed and a typical insulator is taken as an example to analyze and calculate its resistance. Also, the theoretical resistance has been confirmed by numerical simulation using COMSOL Multiphysics software.

In the literature, leakage current monitoring is used to test different types of insulators, including single hanging insulators, terminal insulators, insulators installed in posts, insulators Anti-pollution and fog, insulators with an umbrella or on an insulator chain, etc. have been implemented [19].

Among the various insulator monitoring algorithms, leakage current stands out as one of the most meaningful indicators of contamination performance, as it indicates how close the string of insulators is to flashover. Reference [20] presents a new method for predicting the leakage current in insulator strings by considering the weather information of the insulators. The analysis is based on a combination of a newly developed Cumulative Pollution Index (CPI), which estimates the deposition of dissolved pollution in the insulator strings, and a machine learning technique such as the Random Forest algorithm.

One of the main problems in monitoring the insulator based on its leakage current is the analysis of the results of the measured leakage current under varying harmonic content in the system voltage. Based on this, reference [21] states the possibility of using the instantaneous value of the time integral of the leakage current as a parameter with low sensitivity to monitor the leakage current in the presence of voltage harmonics in insulators.

Also, in [22], an innovation has been proposed to identify the health condition of composite insulators by examining leakage current along with electric field stress (EFS) and electric potential (EP) profiles. It should also be noted that the deposition of pollution on the surface of polymer insulator is a serious issue because it often leads to flashover and even insulator failure. Accordingly, in [23], to estimate the intensity of the contamination level, the surface leakage current (SLC) signals of a polymer insulator with a contaminated surface have been analyzed in the time-frequency domain through the hyperbolic stack transform (HST).

Due to high voltage in substations, manual inspection of insulators can be very dangerous. Therefore, in [24], a non-invasive computer vision system based on an infrared thermal camera (IRT) is presented for automatic monitoring and visual inspection of overhead electrical insulator. Also, reference [25] has also used deep learning to condition monitoring on the insulator.

Distribution transformers maintenance scheduling using Artificial Intelligence (AI) has been investigated according to [26]. Also, the improved relay algorithm for detecting and classifying transmission line faults in HVDC unipolar transmission system using the transient energy signal function has been investigated in [27]. This procedure has been improved in [28] by testing and fault detecting during the operation of HV power cables.

According to the literature review, Table 1 shows the comparison of the previous and proposed methods. According to this table, data monitoring, contamination, failure prediction, flashover, maintenance, classification, machine learning, and image recognition have been evaluated in previous works. Data monitoring, contamination, failure prediction, flashover, and maintenance have been considered similar to the previous works in this paper. It should be noted that these references have been selected according to failure detection in insulators/or modeling of failure prediction and life estimation. Therefore, the intended reference may not be about the insulator, although the failure prediction model is compared with this work.

**Table 1:** Comparison of the previous and proposed methods.

No.	Aspect Consideration	[1]	[3]	[7]	[8]	[13]	[17]	[18]	[22]	[23]	[24]	[25]	[26]	[28]	This work
1	Data Monitoring	✓	✓	✓	✓		✓	✓	✓	✓	✓	✓	✓	✓	✓
2	Contamination	✓	✓			✓	✓	✓	✓	✓			✓		✓
3	Failure Prediction		✓	✓	✓			✓			✓	✓	✓	✓	✓
4	Flashover	✓				✓									✓
5	Maintenance		✓									✓	✓		✓
6	Classification	✓								✓	✓	✓		✓	
7	Machine Learning	✓		✓	✓						✓	✓	✓		
8	Image Recognition			✓	✓										
9	Failure Probability														✓
10	Failure Location														✓
11	Failure level														✓

Also, according to Table 1, the contribution of this paper are failure probability, failure location and failure level detection. Based on this, by having the leakage current data and the characteristics of these insulator data, the probability function, the range of health and failure of each data as well as its health grade are obtained, and after that the probability of failure and maintenance decision-making will be expressed according to the proposed methodology. Finally, the location and type of damaged part in the insulator will be determined.

Therefore, considering the importance of insulators in transmission lines and the vulnerability of these equipment to failure, it will be very important to identify the failure rate of these equipment in the network and notify the maintenance team with the purpose of inspecting these equipment. The model proposed in this paper is implemented on the data available in [1]. It should be noted that this data is not related to the damaged insulator, because the purpose of this paper is to analyze the data obtained from the insulator to detect the time of failure and warn the maintenance team. Therefore, the maintenance team is not supposed to improve the damaged insulator, but by analyzing the data obtained from the insulator, estimates the damage of the insulator and chooses the worse equipment.

The rest of this paper is constructed as follows. In Section 2, the basic tools such as leakage current, probability function, border area, and health grade identification will be introduced. In Section 3, the proposed methodology such as failure probability, maintenance decision-making (MDM), and cumulative MDM will be introduced. In Section 4 and 5, the results and discussion will be presented and finally, the paper concludes in Section 5.

## 2. Basic tools

In this section, leakage current, desired failure probability function, border area identification, and health grade identification will be stated.

### 2.1. Leakage current

**Fig 1:**
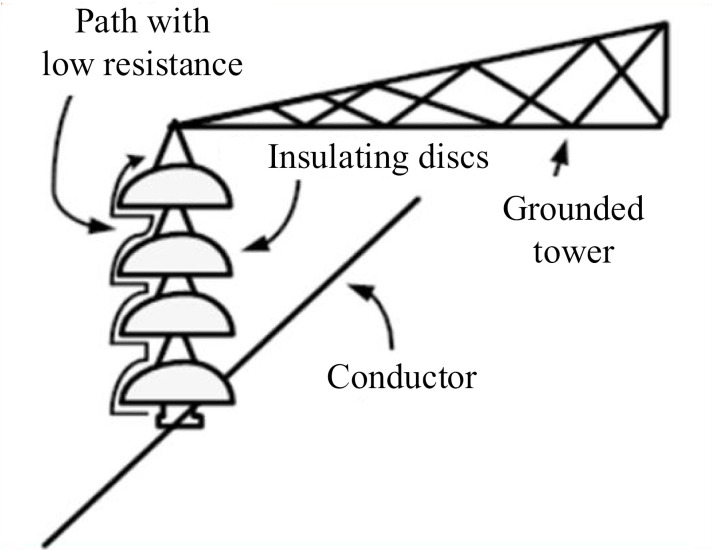
Insulator with contamination and LC.

In a simple definition, any current that flows from a hot conductor to the ground on the external surface of a device is called LC. In the discussion of insulators used in distribution and sub-transmission lines, the current that flows on the surface of the insulator is called insulator LC. The amount of LC of the insulator is one of the very important factors that must be taken into account in the design of the insulator and its installation in the lines. Although this current cannot enter the insulator, it has an effect on the performance of the insulator and flows on a path with low relative resistance on the surface of the insulator. This path with low resistance is actually the interface between the insulator and the surrounding air. Fig 1 shows a schematic of the LC path on an insulator.

It is also necessary to mention that in this article, the input data of the insulator is needed and based on these data and the proposed model, the condition of the insulator failure is estimated. In other words, the monitored data of the equipment that expresses its condition is important, and this equipment can be any type of insulation.

The properties of this characteristic can be derived in two time and frequency domains of the LC signal. Six features/LC indices in each time and frequency domains have been extracted in this work, which will be explained below.

#### 2.1.1. Leakage current monitoring.

LC Monitoring and voltage characteristics in insulators of transmission lines is considered as a good technique to predict the physical condition of insulators. According to [1], the time and frequency domain characteristics of LC in different situations were extracted to evaluate the health condition of the insulator. The intensity of pollution shown by the following factors depends on the insulation:

- Soluble Deposit Density (SDD),
- Wet surface (Wt),
- Non-Soluble Deposit Density (NSDD)
- Uneven distribution of pollution (Pu/PL)

The time domain characteristics of the LC signal include the following:

- LC signal peak (C_1_),
- Phase shift between applied voltage and LC (C_2_),
- LC signal slope between two consecutive peaks (C_3_) and
- Crown factor (C_4_)

The frequency domain characteristics of the LC signal include the following:

- Total harmonic distribution (C_5_)
- Harmonic ratio index (C_6_),

Then the LC indices were classified based on the results of laboratory tests to reflect the physical condition of the insulators. The findings showed that the proposed indicators have an important effect in determining the physical condition of insulators [1]. To verify these indicators, it is necessary to place these items in different groups introduced according to the IEC 60507 standard. Based on this, the SSD index is defined as follows [1]:


SDD=5.7×σ201.03×VA (1)

where σ_20_ represents the conductivity of the pollution solution at 20 degrees Celsius, V represents the volume of the pollution solution and A is the insulation surface area. Meanwhile, (2) is used to determine NSDD [1]:


NSDD=wS−wi×103A (2)

where w_s_ is the mass of filter paper under contamination and w_i_ is the mass of filter paper in dry conditions. As shown in Table 2, in this study three degrees of SDD and NSDD have been evaluated according to light pollution, medium and high (according to IEC 60507 standard) [1]:

**Table 2:** Pollution severity readings.

Parameter	Value			
Contamination Level	Clean	Light	Medium	Heavy
SDD (mg/cm2)	0.00	0.05	0.12	0.20
NSDD (mg/cm2)	0.00	0.15	0.25	0.35
Wt (1/h)	0	3	6	9

#### 2.1.2. Leakage current characteristics.

In this part of this paper, the leakage current characteristics are analyzed in time and frequency domains.

2.1.2.1. Time domain LC characteristics: LC peak (I_m_) and phase shift between the applied voltage and LC (φ) are obtained from the general AC formula and according to (3) [1]:


$$I=I_{m}\cdot sin\left(\omega t+\phi \right)$$ (3)

where ω is the angular frequency calculated with ω=2πf and f = 50 Hz. Now, the characteristics of C_1_ and C_2_ can be obtained as the following equation [1]:


$$C_{1}=I_{m}$$ (4)

$$C_{2}=\phi =\frac{\Delta t}{T}\times 360{}^{\circ}$$ (5)

Calculating the slope of the line between two successive peaks of the leakage current signal has obtained the third property of C_3_. So [1]:


$$C_{3}=\frac{\sum_{n=1}^{m}\left|y_{n}-y_{n-1}\right|}{x_{n}-x_{n-1}}=\frac{\sum_{n=1}^{m}\left|\Delta y_{n}\right|}{\Delta x_{n}}$$ (6)

where ∆ y_n_ is the LC difference between neighboring peaks in n and ∆ x_n_ is the time period between these peaks. The fourth feature is the crest coefficient (C_4_), which is calculated by dividing the peak value by the RMS value of the leakage current. As a result, the following equation expresses the characteristic of C_4_ [1]:


$$C_{4}=\frac{I_{Peak}}{I_{RMS}}$$ (7)

2.1.2.2. Frequency domain LC characteristics: Considering that the frequency range of LC below 500 Hz has characteristic features for insulators under pollution, the odd harmonics and THD of LC below 500 Hz have been used to provide indicators to evaluate the condition of insulators. LC frequency characteristics are expressed by C_5_ (THD) and C_6_ (harmonic ratio) indices and according to (8) and (9) [1]:


$$C_{5}=THD=\frac{\sqrt{\sum_{n=2}^{\infty }I_{n}}}{I_{1}}$$ (8)

$$C_{6}=\frac{\sum_{n}I_{n}}{I_{3}},n=5,7,9$$ (9)

where n represents the odd harmonic order.

### 2.2. Probability function

The reason for using normal distribution in insulator LC data is that:

1- The data obtained from the insulator leakage current is caused by physical factors.
2- These values depend on the natural condition of the equipment, and this natural condition is also dependent on environmental factors and health or damage of the equipment.

Therefore, the expression of normal distribution for insulator LC is justified. The probability distribution calculation based on the normal function for the insulator LC parameters is such that the historical information of the LC is received first. Then, according to the received data, the normal distribution model for each parameter is obtained as (10).


$$X\approx N\left(\mu^{ds},{\left(\sigma^{ds}\right)}^{2}\right)$$ (10)

In the above equation, µ  is the mean, σ is the standard deviation of the data, and ds is the dataset. The above normal distribution is calculated based on (11).


$$f\left(x,\mu^{ds},{\left(\sigma^{ds}\right)}^{2}\right)=\frac{1}{\sqrt{2\pi {\left(\sigma^{ds}\right)}^{2}}}e^{-\frac{{\left(x-\mu^{ds}\right)}^{2}}{2{\left(\sigma^{ds}\right)}^{2}}},forx=C_{j}$$ (11)

So, the cumulative normal distribution is based on (12):


$$F\left(x,\mu^{ds},{\left(\sigma^{ds}\right)}^{2}\right)=\frac{1}{\sqrt{2\pi {\left(\sigma^{ds}\right)}^{2}}}\int_{-\infty }^{x}e^{-\frac{{\left(\tau -\mu^{ds}\right)}^{2}}{2{\left(\sigma^{ds}\right)}^{2}}}d\tau ,forx=C_{j}$$ (12)

**Fig 2:**
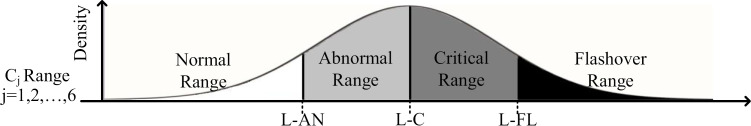
Leakage current data condition on normal distribution.

By receiving new data, the normal distribution is updated in each step and the new µ  and σ values are calculated. Then, in each step and according to the limits specified in Fig 2, the probability value of Normal, Abnormal, Critical and Pre-flashover probability will be obtained.

So that the behavior of the indicators is defined in Table 3, where L_AN, L_C, and L_FL are the lower- Abnormal, lower-Critical, and lower-Pre-flashover values, respectively.

**Table 3:** Definition of indices behavior.

Index	Range			
	Normal	Abnormal	Critical	Pre-Flashover
Cj	<L_AN	>L_AN & < L_C	>L_C & < L_FL	>L_FL

### 2.3. Border area identification

**Fig 3:**
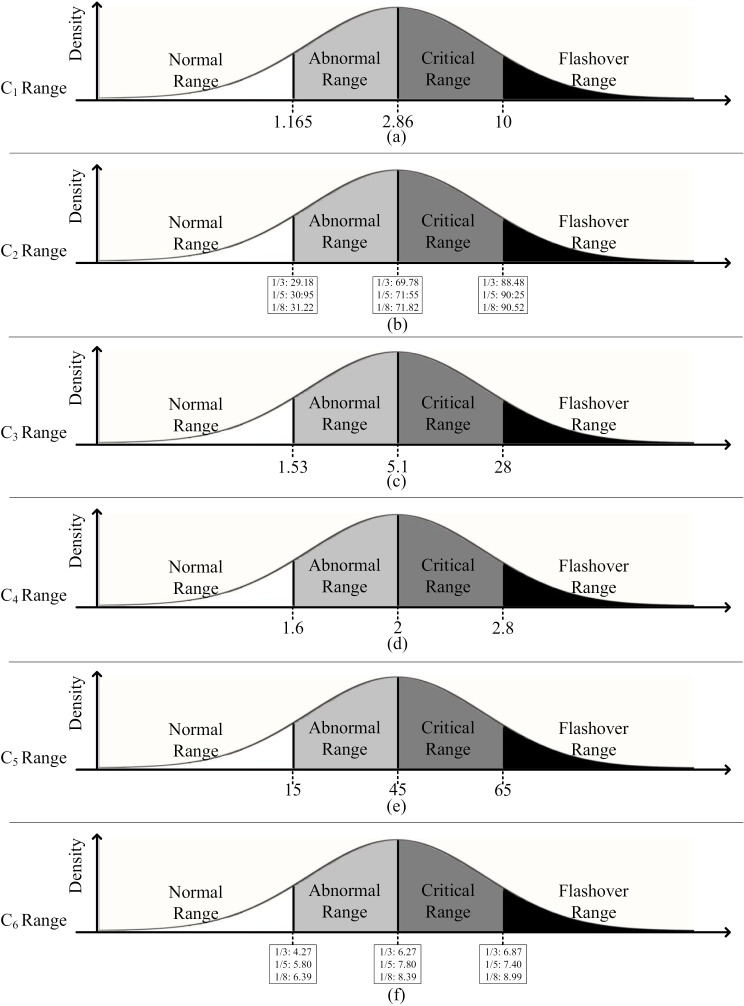
Border area scaling of C_2_-C_6_ in normal distribution.

In this section, the border ranges of each of the criteria C_1_-C_6_ will be discussed. Normal, Abnormal, Critical and Pre-flashover boundary ranges of each of the criteria C_1_-C_6_ are shown in Fig 3 [1]. The limitations in this paper depend on the type of pollution and the type of insulators. These values have been determined by various tests and according to the data obtained by expert teams and are determined in [1] and [29].

### 2.4. Health grade identification

Assuming that the cumulative normal distribution in (12) is used to represent the health grade of an object/mechanism, the four states are (i) Normal, (ii) Abnormal, (iii) Critical and (iv) Pre-flashover condition, can be attributed to the bell function shown in Fig 2. In this representation, being in the abnormal range indicates that there may be minor damage and minor failure in the object.

Reaching the critical range indicates that the object cannot work properly and has a major failure. This condition is a warning to the maintenance team to evaluate the condition of the equipment before disaster strikes. Therefore, the equipment should be subject to preventive maintenance in order to avoid more serious risks. In this paper, the monitored data of the insulator has been used so that the maintenance team can identify the insulator that is capable of failure before the occurrence of an accident and failure in the insulator and damage of the electrical network.

Finally, if the condition of the object falls within the pre-flashover range, it means that there is a failure and the object/mechanism cannot function properly and immediate repair/replacement decisions must be made.

Now for the object under consideration (C_j_), for a given data set (ds):

i. The probability of being in the normal range will be as:

$$P_{C_{j}}^{ds}\left(N\right)=F_{Cj}^{ds}\left({\left(L-AN\right)}_{C_{j}},\mu^{ds},{\left(\sigma^{ds}\right)}^{2}\right)$$ (13)

ii. The probability of being in the abnormal range will be as:

$$P_{C_{j}}^{ds}\left(AN\right)=F_{Cj}^{ds}\left({\left(L-C\right)}_{C_{j}},\mu^{ds},{\left(\sigma^{ds}\right)}^{2}\right)-F_{C_{j}}^{ds}\left({\left(L-AN\right)}_{C_{j}},\mu^{ds},{\left(\sigma^{ds}\right)}^{2}\right)$$ (14)

iii. The probability of being in the critical range will be as:

$$P_{C_{j}}^{ds}\left(C\right)=F_{Cj}^{ds}\left({\left(L-FL\right)}_{C_{j}},\mu^{ds},{\left(\sigma^{ds}\right)}^{2}\right)-F_{C_{j}}^{ds}\left({\left(L-C\right)}_{C_{j}},\mu^{ds},{\left(\sigma^{ds}\right)}^{2}\right)$$ (15)

iv .The probability of being in the pre-flashover range will be as:

$$P_{C_{j}}^{ds}\left(FL\right)=1-F_{Cj}^{ds}\left({\left(L-FL\right)}_{C_{j}},\mu^{ds},{\left(\sigma^{ds}\right)}^{2}\right)$$ (16)

## 3. Proposed methodology

**Fig 4:**
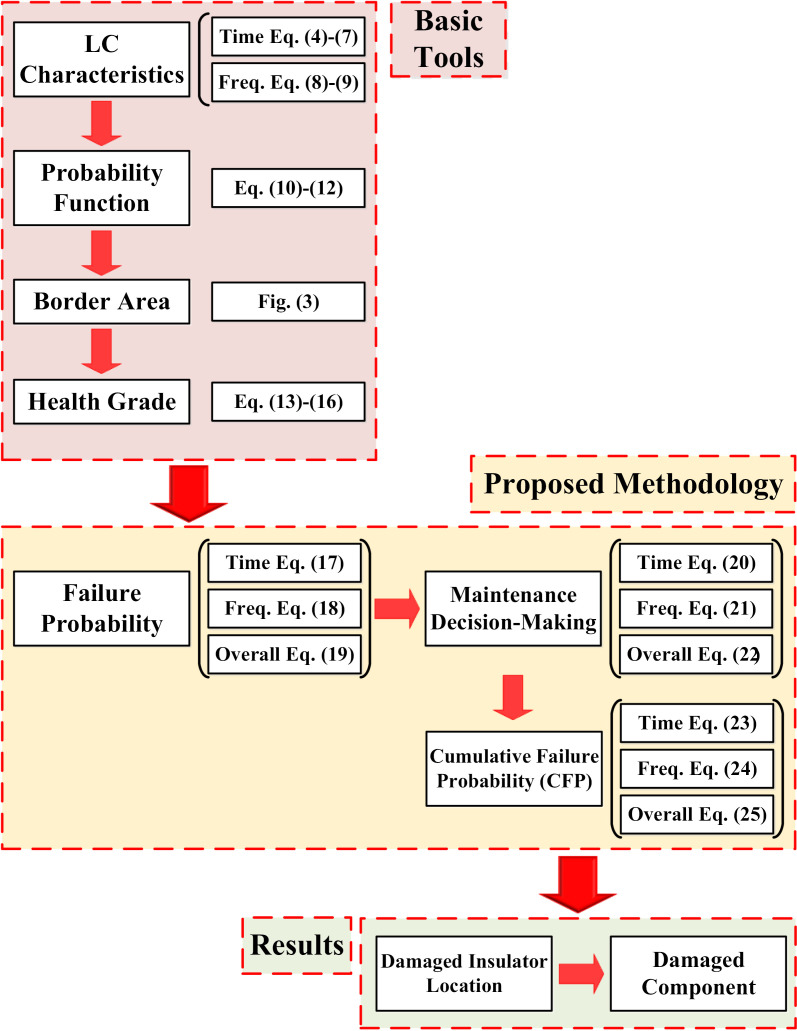
Proposed Methodology Flowchart.

In this section, failure probability, maintenance decision-making, and cumulative failure probability identification will be stated. The process of this proposed methodology flowchart is shown in Fig 4.

### 3.1. Failure probability identification

In this section, the failure probability of the time domain, frequency domain and overall conditions in the insulator is introduced.

a) The probability of time domain characteristic being in the normal, abnormal, critical and pre-flashover intervals can be defined as:

$$\begin{array}{l} P_{T}^{ds}\left(N\right)=\overset{4}{\underset{j=1}{\Pi }}\left(P_{C_{j}}\left(N\right)\right)\\ P_{T}^{ds}\left(AN\right)=\overset{4}{\underset{j=1}{\Pi }}\left(P_{C_{j}}\left(AN\right)\right)\\ P_{T}^{ds}\left(C\right)=\overset{4}{\underset{j=1}{\Pi }}\left(P_{C_{j}}\left(C\right)\right)\\ P_{T}^{ds}\left(FL\right)=\overset{4}{\underset{j=1}{\Pi }}\left(P_{C_{j}}\left(FL\right)\right) \end{array}$$ (17)

b) The probability of frequency domain characteristic being in the normal, abnormal, critical and pre-flashover intervals can be defined as:

$$\begin{array}{l} P_{F}^{ds}\left(N\right)=\overset{6}{\underset{j=5}{\Pi }}\left(P_{C_{j}}\left(N\right)\right)\\ P_{F}^{ds}\left(AN\right)=\overset{6}{\underset{j=5}{\Pi }}\left(P_{C_{j}}\left(AN\right)\right)\\ P_{F}^{ds}\left(C\right)=\overset{6}{\underset{j=5}{\Pi }}\left(P_{C_{j}}\left(C\right)\right)\\ P_{F}^{ds}\left(FL\right)=\overset{6}{\underset{j=5}{\Pi }}\left(P_{C_{j}}\left(FL\right)\right) \end{array}$$ (18)

c) The probability of overall characteristic being in the normal, abnormal, critical and pre-flashover intervals can be defined as:

$$\begin{array}{l} P_{OV}^{ds}\left(N\right)=\overset{6}{\underset{j=1}{\Pi }}\left(P_{C_{j}}\left(N\right)\right)\\ P_{OV}^{ds}\left(AN\right)=\overset{6}{\underset{j=1}{\Pi }}\left(P_{C_{j}}\left(AN\right)\right)\\ P_{OV}^{ds}\left(C\right)=\overset{6}{\underset{j=1}{\Pi }}\left(P_{C_{j}}\left(C\right)\right)\\ P_{OV}^{ds}\left(FL\right)=\overset{6}{\underset{j=1}{\Pi }}\left(P_{C_{j}}\left(FL\right)\right) \end{array}$$ (19)

### 3.2. Maintenance decision-making identification

**Fig 5:**
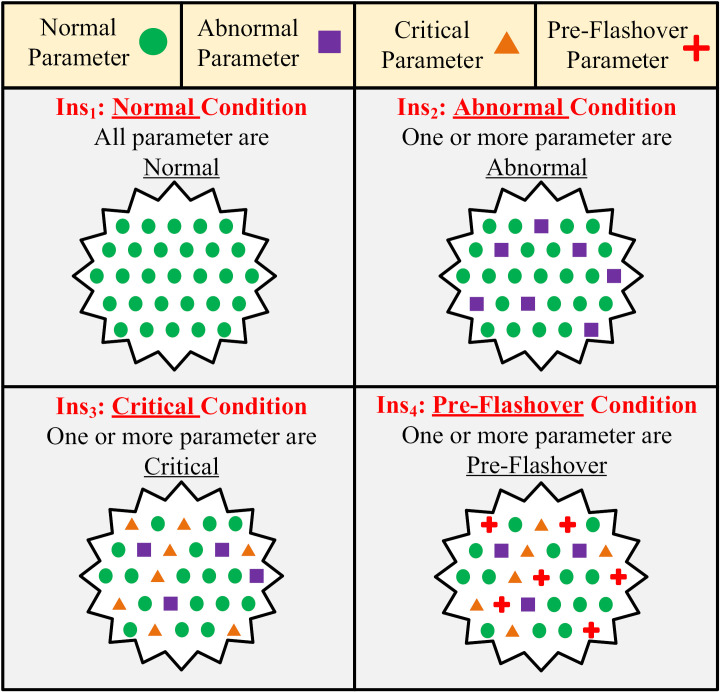
Summary schematic of condition rule.

Now, given the normal distribution, to finalize the insulator conditions for a data set, i.e., to find the probability of normal, abnormal, critical and/or pre-flashover mode, the following rule applies:

i. The insulator will accept the worse condition, even if all but one of the attributes have better conditions, the insulator will accept that worse condition. For example, if there is only one temporal feature in the abnormal condition, the insulator condition for that data set will be abnormal. Accordingly, if only all the properties are within the normal range, the insulator condition will be normal. The summary schematic of this rule is shown in Fig 5. According to this figure, by observing one or more parameters in a specific failure level (Abnormal, Critical or Pre-Flashover), the insulator is placed in the same failure level.

Based on the above-mentioned rule:

a) The maintenance decision-making of time features condition being in the pre-flashover, critical, abnormal, and normal intervals can be defined as:

$$\begin{array}{l} FP_{T,FL}^{ds}=1-\overset{4}{\underset{j=1}{\Pi }}\left(P_{C_{j}}^{ds}\left(N\right)+P_{C_{j}}^{ds}\left(AN\right)+P_{C_{j}}^{ds}\left(C\right)\right)\\ FP_{T,C}^{ds}=1-FP_{T,FL}^{ds}-\overset{4}{\underset{j=1}{\Pi }}\left(P_{C_{j}}^{ds}\left(N\right)+P_{C_{j}}^{ds}\left(AN\right)\right)\\ FP_{T,AN}^{ds}=1-FP_{T,FL}^{ds}-FP_{T,C}^{ds}-\overset{4}{\underset{j=1}{\Pi }}\left(P_{C_{j}}^{ds}\left(N\right)\right)\\ FP_{T,N}^{ds}=\overset{4}{\underset{j=1}{\Pi }}\left(P_{C_{j}}^{ds}\left(N\right)\right) \end{array}$$ (20)

b) The maintenance decision-making of frequency features condition being in the pre-flashover, critical, abnormal, and normal intervals can be defined as:

$$\begin{array}{l} FP_{F,FL}^{ds}=1-\overset{6}{\underset{j=5}{\Pi }}\left(P_{C_{j}}^{ds}\left(N\right)+P_{C_{j}}^{ds}\left(AN\right)+P_{C_{j}}^{ds}\left(C\right)\right)\\ FP_{F,C}^{ds}=1-FP_{T,FL}^{ds}-\overset{6}{\underset{j=5}{\Pi }}\left(P_{C_{j}}^{ds}\left(N\right)+P_{C_{j}}^{ds}\left(AN\right)\right)\\ FP_{F,AN}^{ds}=1-FP_{T,FL}^{ds}-FP_{T,C}^{ds}-\overset{6}{\underset{j=5}{\Pi }}\left(P_{C_{j}}^{ds}\left(N\right)\right)\\ FP_{F,N}^{ds}=\overset{6}{\underset{j=5}{\Pi }}\left(P_{C_{j}}^{ds}\left(N\right)\right) \end{array}$$ (21)

c) The maintenance decision-making of overall condition being in the pre-flashover, critical, abnormal, and normal intervals can be defined as:

$$\begin{array}{l} FP_{OV,FL}^{ds}=1-\overset{6}{\underset{j=1}{\Pi }}\left(P_{C_{j}}^{ds}\left(N\right)+P_{C_{j}}^{ds}\left(AN\right)+P_{C_{j}}^{ds}\left(C\right)\right)\\ FP_{OV,C}^{ds}=1-FP_{T,FL}^{ds}-\overset{6}{\underset{j=1}{\Pi }}\left(P_{C_{j}}^{ds}\left(N\right)+P_{C_{j}}^{ds}\left(AN\right)\right)\\ FP_{OV,AN}^{ds}=1-FP_{T,FL}^{ds}-FP_{T,C}^{ds}-\overset{6}{\underset{j=1}{\Pi }}\left(P_{C_{j}}^{ds}\left(N\right)\right)\\ FP_{OV,N}^{ds}=\overset{6}{\underset{j=1}{\Pi }}\left(P_{C_{j}}^{ds}\left(N\right)\right) \end{array}$$ (22)

### 3.3. Cumulative failure probability identification

At the end, the failure probability of each insulator or each time or frequency domain is determined and then the Cumulative Failure Probability (CFP) is determined based on (23)–(25) for time domain, frequency domain, and overall condition of insulator, respectively. The larger this index is, the worse the condition of insulator failure.


$$CFP_{T,FL}=\sum_{ds=1}^{n}FP_{T,FL}^{ds}$$ (23)

$$CFP_{F,FL}=\sum_{ds=1}^{n}FP_{F,FL}^{ds}$$ (24)

$$CFP_{OV,FL}=\sum_{ds=1}^{n}FP_{OV,FL}^{ds}$$ (25)

where ds is the number of datasets related to the insulator.

## 4. Results

In this section, the results related to the condition monitoring of insulator using LC data will be analyzed.

Based on this

A. At first, the results of the proposed procedure for failure estimation of the insulator will be expressed based on the following situations:
  i. Wt indicator

- Failure estimation based on the value of each index (Wt = 0-3-6-9)

ii. *SDD indicator*
  - Failure estimation based on the value of each index (SDD = 0.05-0.12-0.2)
iii. *NSDD indicator*
  - Failure estimation based on the value of each index (NSDD = 0.15-0.25-0.35)

B. Then, MDM comparison in overall condition is done according to the following modes:
  i. *Wt modes*
    a) For time do domain, frequency domain, and overall condition
    b) For Wt = 0-3-6-9
  ii. SDD modes
    a) For time do domain, frequency domain, and overall condition
    b) For SDD = 0.05-0.12-0.2
  iii. NSDD modes
    a) For time do domain, frequency domain, and overall condition
    b) For NSDD = 0.15-0.25-0.35

Now each of these situations will be evaluated.

### 4.1. Failure estimation

According to this section, the results of failure estimation will be expressed in three Wt, SDD, and NSDD indicators.

#### 4.1.1. Failure estimation for WT indicator.

**Fig 6:**
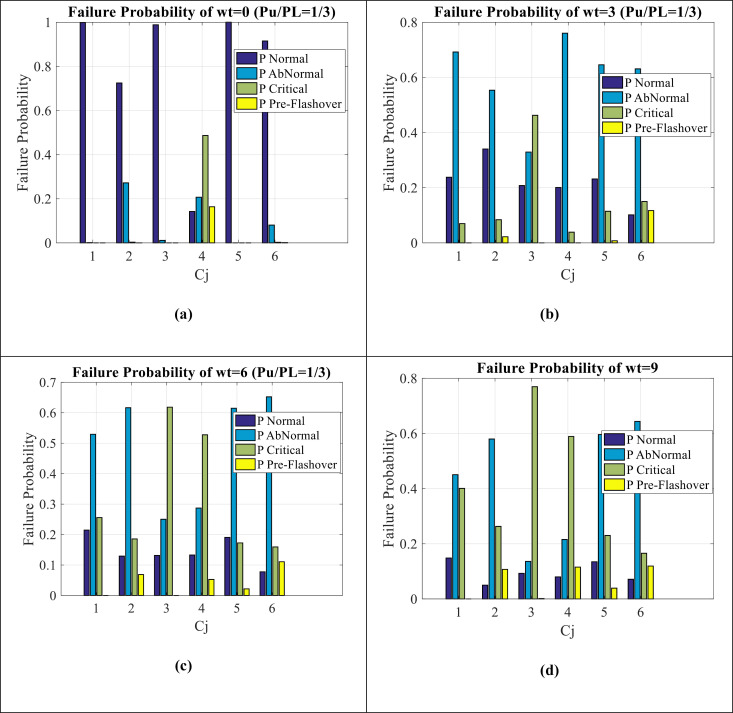
Failure estimation of Wt indicator (a) **Wt = 0** (b) **Wt = 3** (c) **Wt = 6** (d) **Wt = 9.**

Fig 6 (a–d) show failure estimation for Wt = 0, Wt = 3, Wt = 6, and Wt = 9, respectively. It can be seen as follows:

- The failure estimation of all C_1_-C_6_ and for all (Wt = 0-3-6-9) is observed.
- In Wt = 0 condition (Fig (6-a)), normal values are very high and pre-flashover condition is not seen in the results (It has been observed in only C_4_ values).
- As it moves towards Wt = 9 condition (Fig (6-d)), the pre-flashover condition increases and decreases from normal values.
- Most of the data of Wt = 0 is on the normal condition, most of the data of Wt = 3 is on the abnormal condition, most of the data of Wt = 6 is between the abnormal and critical condition, and finally most of the data of Wt = 9 is between the abnormal, critical and pre-flashover condition.

#### 4.1.2. Failure estimation for SDD indicator.

**Fig 7:**
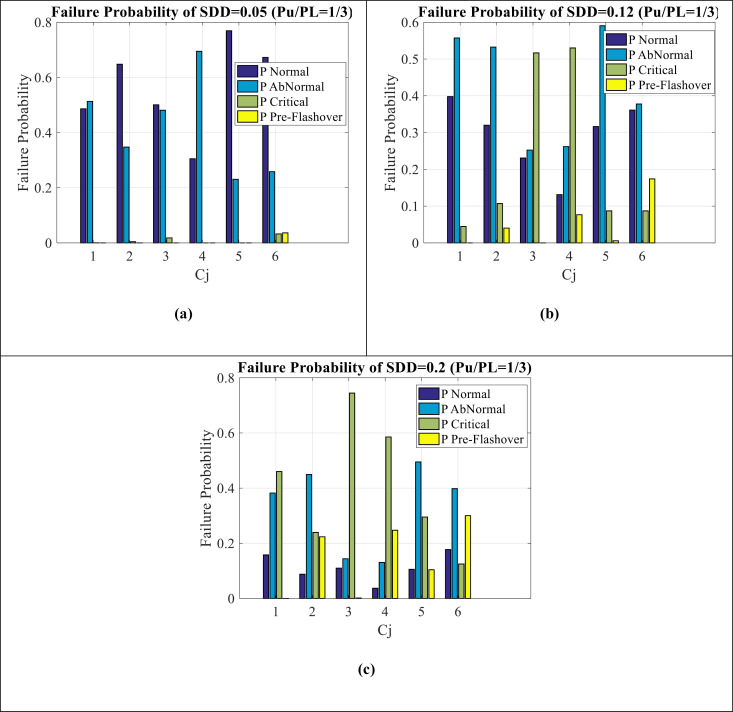
Failure estimation of SDD indicator (a) SDD = 0.05 (b) SDD = 0.12 (c) SDD = 0.2.

Fig 7(a–c) show failure estimation for SDD = 0.05, SDD = 0.12, and SDD = 0.2, respectively. It can be seen as follows:

- The failure estimation of all C_1_-C_6_ and for all (SDD = 0.05-0.12-0.2) is observed.
- In SDD = 0.05 condition (Fig 7(a)), normal and abnormal values are very high and pre-flashover condition is not seen in the results (It has been observed in only C_6_ values).
- As it moves towards SDD = 0.2 condition (Fig 7(c)), the critical and pre-flashover condition increases and decreases from normal values.
- Most of the data of SDD = 0.05 is on the normal and abnormal condition, most of the data of SDD = 0.12 is on the abnormal and critical condition, and finally most of the data of SDD = 0. 2 is between the abnormal, critical and pre-flashover condition.
- It should be noted that the impact of SDD indicator compared to Wt indicator seems to be more in the failure condition.

#### 4.1.3. Failure estimation for NSDD indicator.

**Fig 8:**
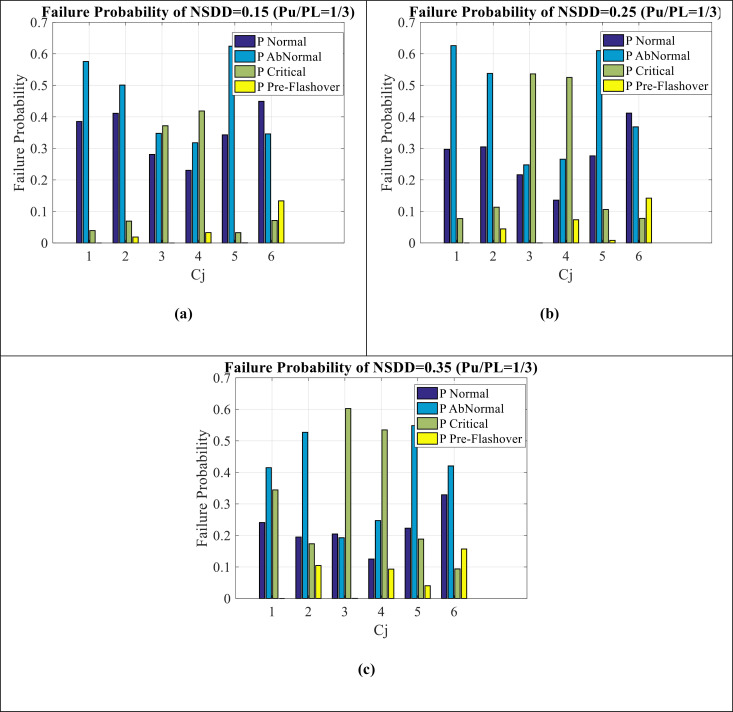
Failure estimation of NSDD indicator (a) **NSDD = 0.15** (b) **NSDD = 0.25** (c) **NSDD = 0.35.**

Fig 8(a–c) show failure estimation for NSDD = 0.15, NSDD = 0.25, and NSDD = 0.35, respectively. It can be seen as follows:

- The failure estimation of all C_1_-C_6_ and for all (NSDD = 0.15-0.25-0.35) is observed.
- In NSDD = 0.15 condition (Fig (8-a)), abnormal values are very high and some pre-flashover condition is seen (pre-flashover condition of C_6_ is higher). This abnormal condition indicates the increase of insulator failure according to this index.
- As it moves towards NSDD = 0.35 condition (Fig (8-c)), the abnormal and critical condition increases and decreases from normal values.
- Most of the data of NSDD = 0.15 is on the normal and abnormal condition, most of the data of NSDD = 0.25 is on the normal, abnormal and critical condition, and finally most of the data of NSDD = 0. 35 is between the abnormal and critical condition.
- It should be noted that the impact of NSDD indicator compared to Wt and SDD indicator seems to be less in the failure condition.

### 4.2. MDM comparison

According to this section, the results of MDM comparison will be expressed in three Wt, SDD, and NSDD modes.

#### 4.2.1. MDM comparison for WT mode.

**Fig 9:**
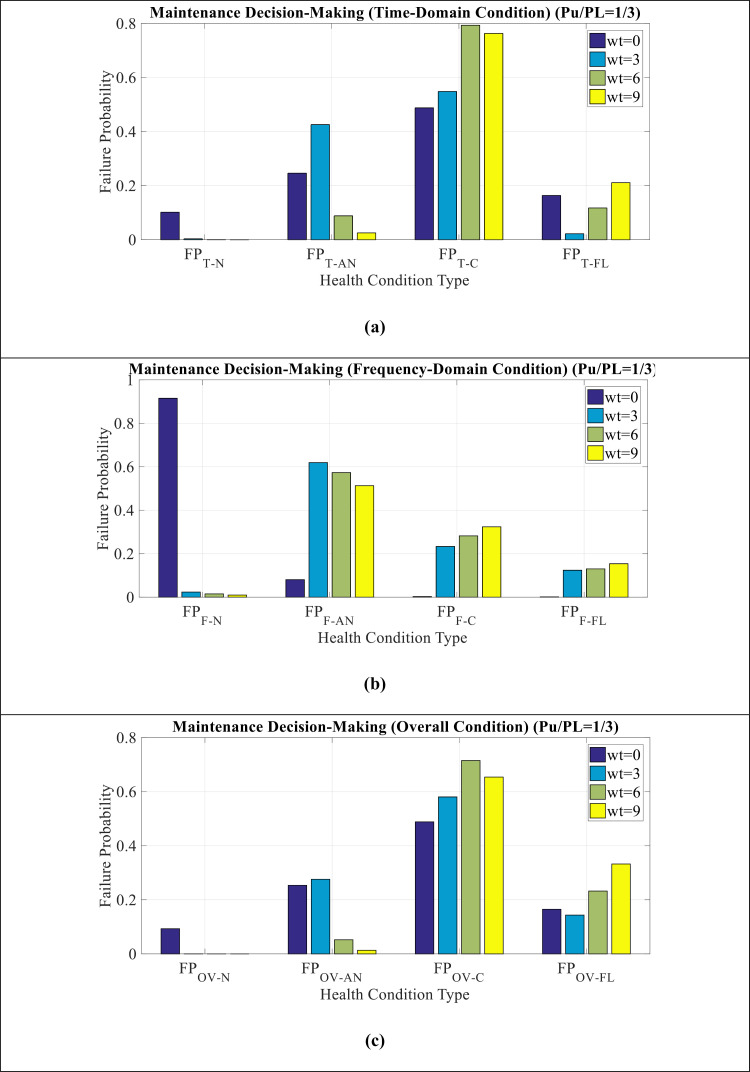
MDM comparison for Wt mode in (a) time domain condition (b) frequency domain condition (c) overall condition.

Fig 9(a–c) show MDM comparison for Wt modes in time domain, frequency domain, and overall condition, respectively. It can be seen as follows:

- Critical condition has the highest values in the time domain. However, in the frequency domain, normal and abnormal condition have more values. In other words, time indicators have a much worse condition than frequency indicators according to Wt mode.
- The overall condition also indicate more value of the critical condition, which is due to the effect of time indicators on this issue.
- The time domain indicators cause the insulator condition to become critical and the frequency domain indicators have suitable conditions. In other words, to investigate the causes of insulator failure, the maintenance team should seek to track the failure of the time domain parameters of the LC.

**Fig 10:**
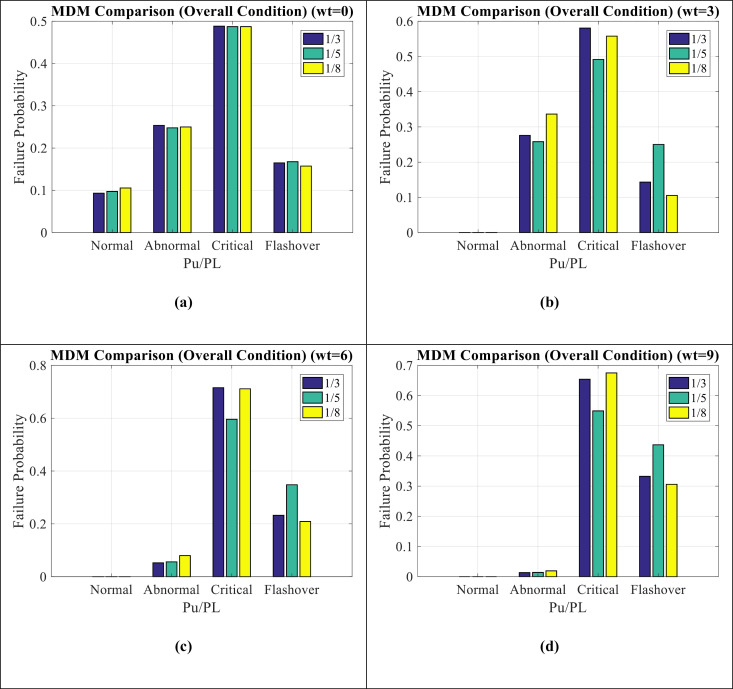
MDM comparison for (a) **Wt = 0** (b) **Wt = 3** (c) **Wt = 6** (d) **Wt = 9.**

Fig 10(a–d) show MDM comparison for Wt = 0, Wt = 3, Wt = 6, and Wt = 9 modes, respectively. It can be seen as follows:

- In WT = 0 condition, the maintenance index shows a critical condition.
- With the increase of Wt from 0 to 9, the critical condition changes to pre-flashover condition, which indicates the worsening of the insulator conditions in this situation.
- The amount of P_U_/P_L_ has not shown a great effect on the amount of insulator failure.

#### 4.2.2. MDM comparison for SDD mode.

**Fig 11:**
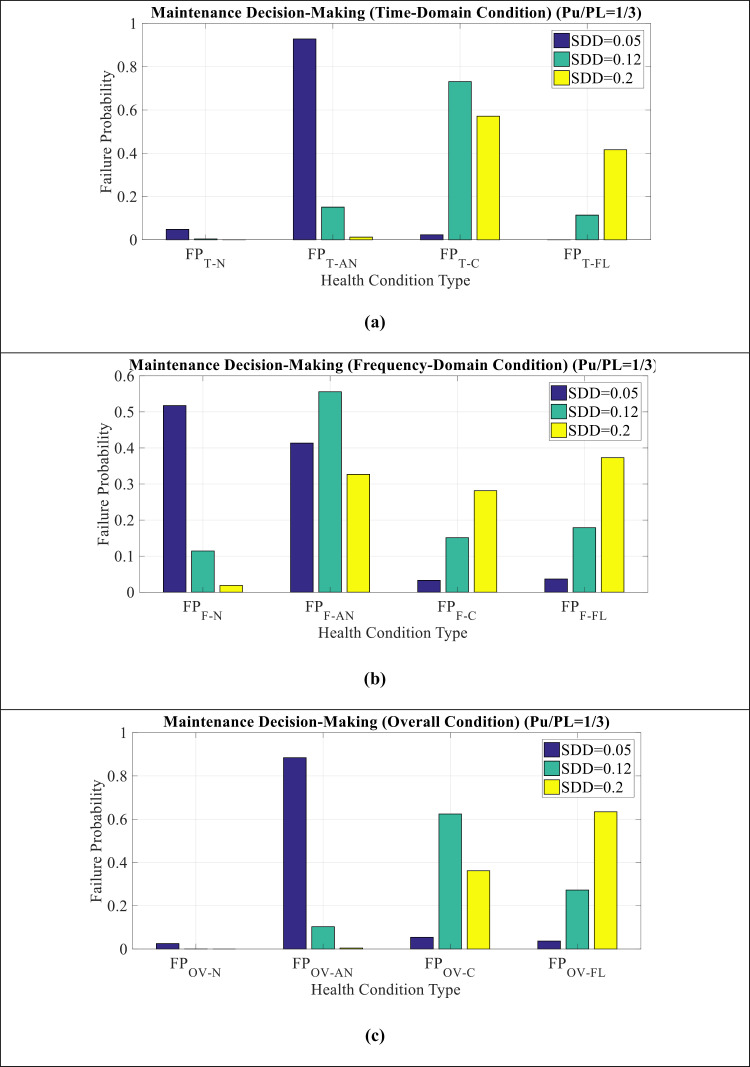
MDM comparison for SDD mode in (a) time domain condition (b) frequency domain condition (c) overall condition.

Fig 11(a–c) show MDM comparison for SDD modes in time domain, frequency domain, and overall condition, respectively. It can be seen as follows:

- Abnormal condition has the highest values in the time domain. However, in the frequency domain, the values are almost in the same range.
- The overall condition also indicate more value of the abnormal condition, which is due to the effect of time indicators on this issue.
- According to these results, it can be seen that it is not mandatory to maintenance the insulator according to SDD mode.

**Fig 12:**
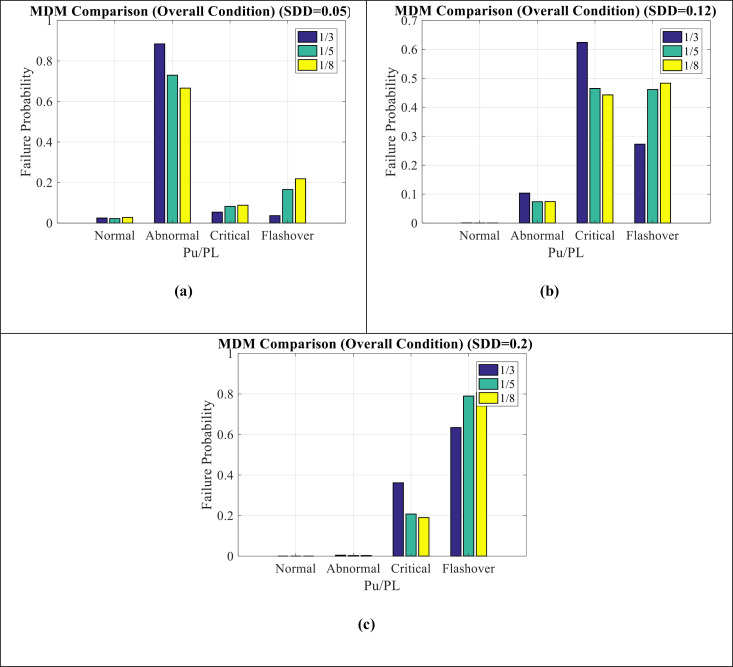
MDM comparison for (a) **SDD = 0.05** (b) **SDD = 0.12** (c) **SDD = 0.2.**

Fig 12(a–c) show MDM comparison for SDD = 0.05, SDD = 0.12, and SDD = 0.2 modes, respectively. It can be seen as follows:

- In SDD = 0.05 condition, the maintenance index shows an abnormal condition.
- With the increase of SDD from 0.05 to 0.2, the abnormal condition changes to pre-flashover condition, which indicates the worsening of the insulator conditions in this situation.
- The amount of P_U_/P_L_ has affected on the results and has led to an increase in the probability of failure in pre-flashover condition and a decrease in the probability of failure in abnormal condition.

#### 4.2.3. MDM comparison for NSDD mode.

**Fig 13:**
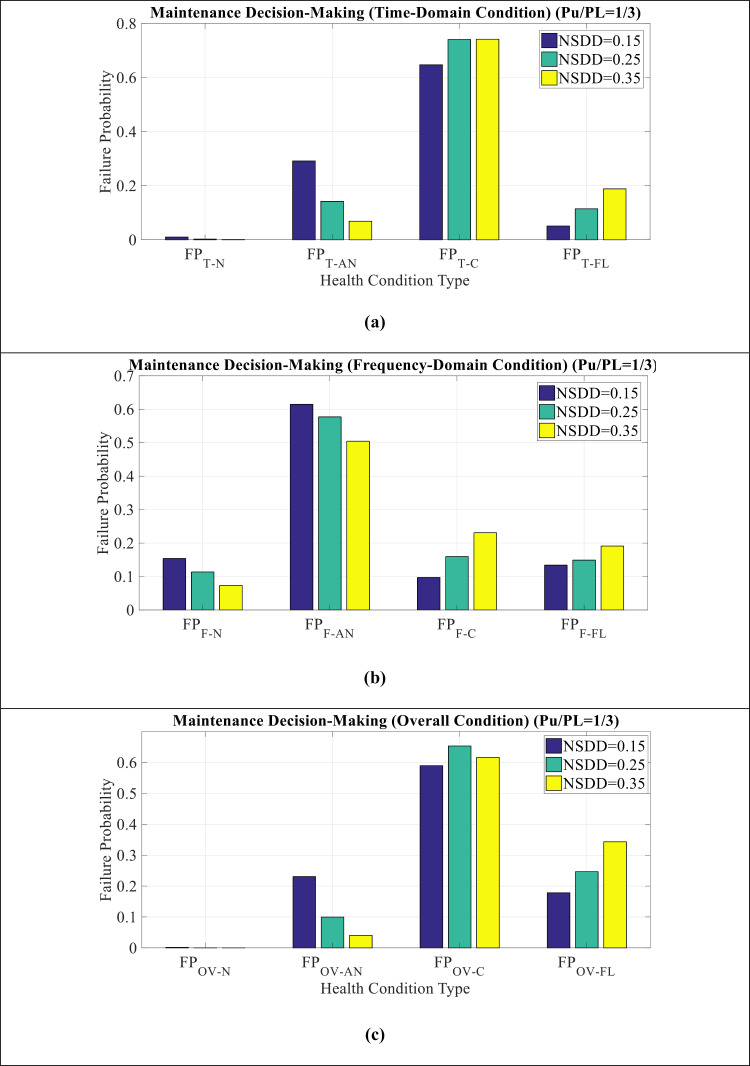
MDM comparison for NSDD mode in (a) time domain condition (b) frequency domain condition (c) overall condition.

Fig 13(a–c) show MDM comparison for NSDD modes in time domain, frequency domain, and overall condition, respectively. It can be seen as follows:

- Critical condition has the highest values in the time domain. However, in the frequency domain, abnormal condition has the highest values.
- The overall condition also indicate more value of the critical condition, which is due to the effect of time indicators on this issue.
- The time domain indicators cause the insulator condition to become critical and the frequency domain indicators have suitable conditions. In other words, to investigate the causes of insulator failure, the maintenance team should seek to track the failure of the time domain parameters of the LC.

**Fig 14:**
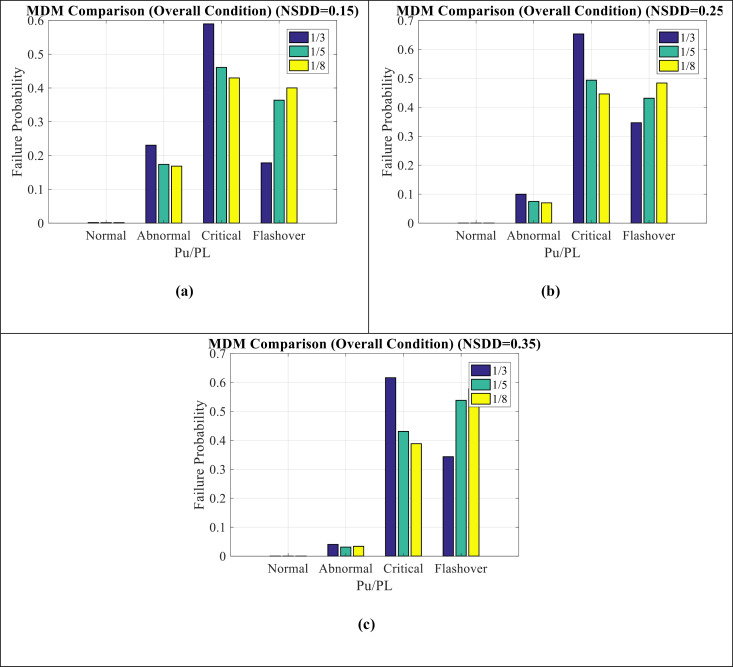
MDM comparison for (a) NSDD = 0.15 (b) NSDD = 0.25 (c) NSDD = 0.35.

Fig 14(a–c) show MDM comparison for NSDD = 0.15, NSDD = 0.25, and NSDD = 0.35 modes, respectively. It can be seen as follows:

- In NSDD = 0.15 condition, the maintenance index shows a critical condition.
- With the increase of NSDD from 0.15 to 0.35, the critical condition changes to pre-flashover condition, which indicates the worsening of the insulator conditions in this situation.
- The amount of P_U_/P_L_ has affected on the results and has led to an increase in the probability of failure in pre-flashover condition and a decrease in the probability of failure in abnormal and critical condition.

## 5. Discussion

In this section, the discussion on the results obtained based on CFP obtained according to equations (23)–(25) will be discussed. Table 4 shows all the results related to failure probability and CFP of all condition.

**Table 4:** Failure probability and CFP of all condition.

Failure Probability	PU/PL	1.3			1.5			1.8			CFP
	SDD	0.05	0.12	0.2	0.05	0.12	0.2	0.05	0.12	0.2	
Time Domain Condition	Normal	4.820	0.390	0.0056	6.240	0. 460	0.006	8.37	0. 500	0.006	0.208
	Abnormal	92.860	15.090	1.230	91.940	15.400	1.500	89.410	15.820	1.780	3.250
	Critical	2.310	73.130	57.140	1.800	72.290	55.680	2.200	72.020	55.770	3.923
	Pre-Flashover	1.400	11.390	41.630	2.100	11.850	42.820	2.200	11.650	42.450	1.618
Frequency Domain Condition	Normal	51.740	11.430	1.870	35.840	6.380	0.770	33.070	7.120	0.880	1.491
	Abnormal	41.330	55.580	32.670	40.770	40.180	17.630	37.850	38.340	16.480	3.208
	Critical	3.270	15.110	28.150	6.860	14.560	18.390	7.260	13.040	16.180	1.228
	Pre-Flashover	3.660	17.880	37.310	16.530	38.880	63.210	21.820	41.500	66.450	3.072
Overall Condition	Normal	2.490	4.400	0.000	2.240	2.900	0.000	2.770	3.600	0.000	0.076
	Abnormal	88.420	10.330	0.430	72.980	7.350	0.280	66.580	7.390	0.310	2.541
	Critical	5.420	62.390	36.170	8.230	46.490	20.760	8.820	44.260	19.000	2.515
	Pre-Flashover	3.670	27.240	63.410	16.550	46.130	78.960	21.840	48.310	80.690	3.868

**Fig 15:**
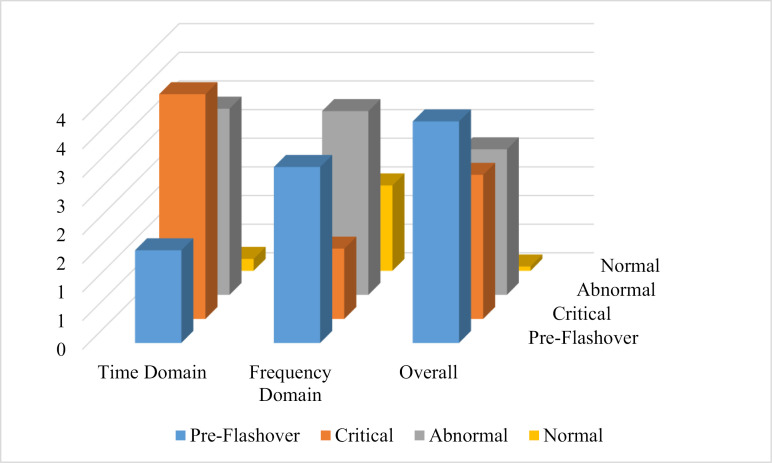
CFP analysis.

According to Fig 15, the results of CFP are also shown. According to this figure:

- The critical situation shows the highest value in the time domain condition. After that, there is an abnormal situation. Also, the pre-flashover and normal states are in the last ranks. Therefore, it can be said that the time domain condition cannot work properly and has a major failure. The maintenance team should examine (C_1_-C_4_) indicators more carefully.
- The abnormal situation shows the highest value in the frequency domain condition. After that, there is a pre-flashover situation. Also, the normal and critical states are in the last ranks. Therefore, it can be said that the frequency domain condition has a minor damage and minor failure. The maintenance team should examine (C_5_-C_6_) indicators with less precision than the time domain condition.
- The pre-flashover situation shows the highest value in the overall condition. After that, there is an abnormal and critical situation. Also, the normal states are in the last ranks with a very low value compared to other indicators. Therefore, it can be said that the overall condition has a fault and failure and the object/mechanism cannot function properly and immediate repair/replacement decisions must be made.

## 6. Conclusion

The function of the insulator in the power system is to isolate different parts of the network from each other, and the health of its operation is very important. In this paper, by using normal distribution, the failure rate of insulator equipment was obtained using LC data. Then, by using these values of the failure rate in the insulator, decision-making for planning the maintenance of the specified equipment and the damaged equipment in the power network were identified. According to this method, the insulator equipment in the power grid will be identified according to the higher failure rate and will be prioritized for maintenance by the relevant team. The proposed method reaches a definite result completely according to specific rules and without the user’s expert opinion. Based on this, the error in its final result will be less and the equipment with failure will be completely distinguished from other equipment. This paper is proposed according to the available data to detect the amount of insulation failure, while the proposed procedure is quite flexible and with more data, a more accurate decision can be reached by this procedure.

## Supporting information

S1 Data Final code. (RAR)

## Abbreviations

AI: Artificial Intelligent
AN: Abnormal
ANN: Artificial Neural Network
C: Critical
CPI: Cumulative Pollution Index
ds: data set
F: Frequency Domain
FL: Pre-Flashover
HVDC: High Voltage Direct Current
LC: Leakage Current
MDM: Maintenance Decision-Making
N: Normal
NSDD: Non-Soluble Deposit Density
OV: Overall Characteristic
Pu/PL: Uneven distribution of pollution
PD: Partial Discharge
R-CNN: Region-based Convolutional Neural Networks
SDD: Soluble Deposit Density
SLC: Surface Leakage Current
T: Time Domain
THD: Total Harmonic Distortion
Wt: Wet Surface
µ: mean
σ: standard deviation
